# Intact Serial Dependence in Schizophrenia: Evidence from an Orientation Adjustment Task

**DOI:** 10.1093/schbul/sbae106

**Published:** 2024-06-27

**Authors:** David Pascucci, Maya Roinishvili, Eka Chkonia, Andreas Brand, David Whitney, Michael H Herzog, Mauro Manassi

**Affiliations:** Laboratory of Psychophysics, Brain Mind Institute, École Polytechnique Fédérale de Lausanne (EPFL), Lausanne, Switzerland; Institute of Cognitive Neurosciences, Free University of Tbilisi, Tbilisi, Georgia; Department of Psychiatry, Tbilisi State Medical University, Tbilisi, Georgia; Laboratory of Psychophysics, Brain Mind Institute, École Polytechnique Fédérale de Lausanne (EPFL), Lausanne, Switzerland; Department of Psychology, University of California, Berkeley, CA, USA; Helen Wills Neuroscience Institute, University of California, Berkeley, CA, USA; Vision Science Group, University of California, Berkeley, CA, USA; Laboratory of Psychophysics, Brain Mind Institute, École Polytechnique Fédérale de Lausanne (EPFL), Lausanne, Switzerland; School of Psychology, University of Aberdeen, King’s College, Aberdeen, UK

**Keywords:** schizophrenia, serial dependence, perceptual priors

## Abstract

**Background and Hypothesis:**

For a long time, it was proposed that schizophrenia (SCZ) patients rely more on sensory input and less on prior information, potentially leading to reduced serial dependence—ie, a reduced influence of prior stimuli in perceptual tasks. However, existing evidence is constrained to a few paradigms, and whether reduced serial dependence reflects a general characteristic of the disease remains unclear.

**Study Design:**

We investigated serial dependence in 26 SCZ patients and 27 healthy controls (CNT) to evaluate the influence of prior stimuli in a classic visual orientation adjustment task, a paradigm not previously tested in this context.

**Study Results:**

As expected, the CNT group exhibited clear serial dependence, with systematic biases toward the orientation of stimuli shown in the preceding trials. Serial dependence in SCZ patients was largely comparable to that in the CNT group.

**Conclusions:**

These findings challenge the prevailing notion of reduced serial dependence in SCZ, suggesting that observed differences between healthy CNT and patients may depend on aspects of perceptual or cognitive processing that are currently not understood.

## Introduction

It has long been proposed that individuals with schizophrenia (SCZ) rely more on sensory input rather than on prior information, which, surprisingly, can lead to more veridical perception compared to healthy controls (CNT).^1–3^ For example, patients with SCZ are less affected by visual illusions that depend on perceptual priors,^4–6^ such as the Hollow-Mask illusion.^2,7–9^ Atypical visual performance in SCZ is often linked to a diminished influence of priors in Bayesian and predictive theories,^10–13^ potentially resulting from deficits in top-down processing domains like attention and working memory.^14–19^ However, empirical evidence is mixed,^20–22^ with several studies also showing intact perceptual illusions in SCZ.^23,24^

Serial dependence, wherein prior stimuli influence current perception,^25–29^ has been recently used as a tool to investigate the influence of prior visual information in SCZ.^30–32^ In tasks where participants are asked to reproduce the feature of a stimulus after a delay, healthy CNT exhibit attractive serial dependence—ie, responses are attracted toward prior stimuli, whereas SCZ patients exhibit a repulsive bias away from the previous stimulus.^30–32^ These findings suggest deficits in integrating perceptual priors—ie, recent stimuli—with current perception in SCZ, which may reflect a general characteristic of the disease. However, this evidence primarily stems from paradigms investigating memory-delayed reproduction tasks with well-visible stimuli, which differ considerably from the classic and widely used orientation adjustment task with weak and masked visual stimuli.^26^ This task typically elicits robust attractive biases in healthy CNT,^27,28^ and thus, it provides a key test to offer complementary evidence of atypical serial dependence using distinct paradigms and stimuli.

**Fig. 1.**
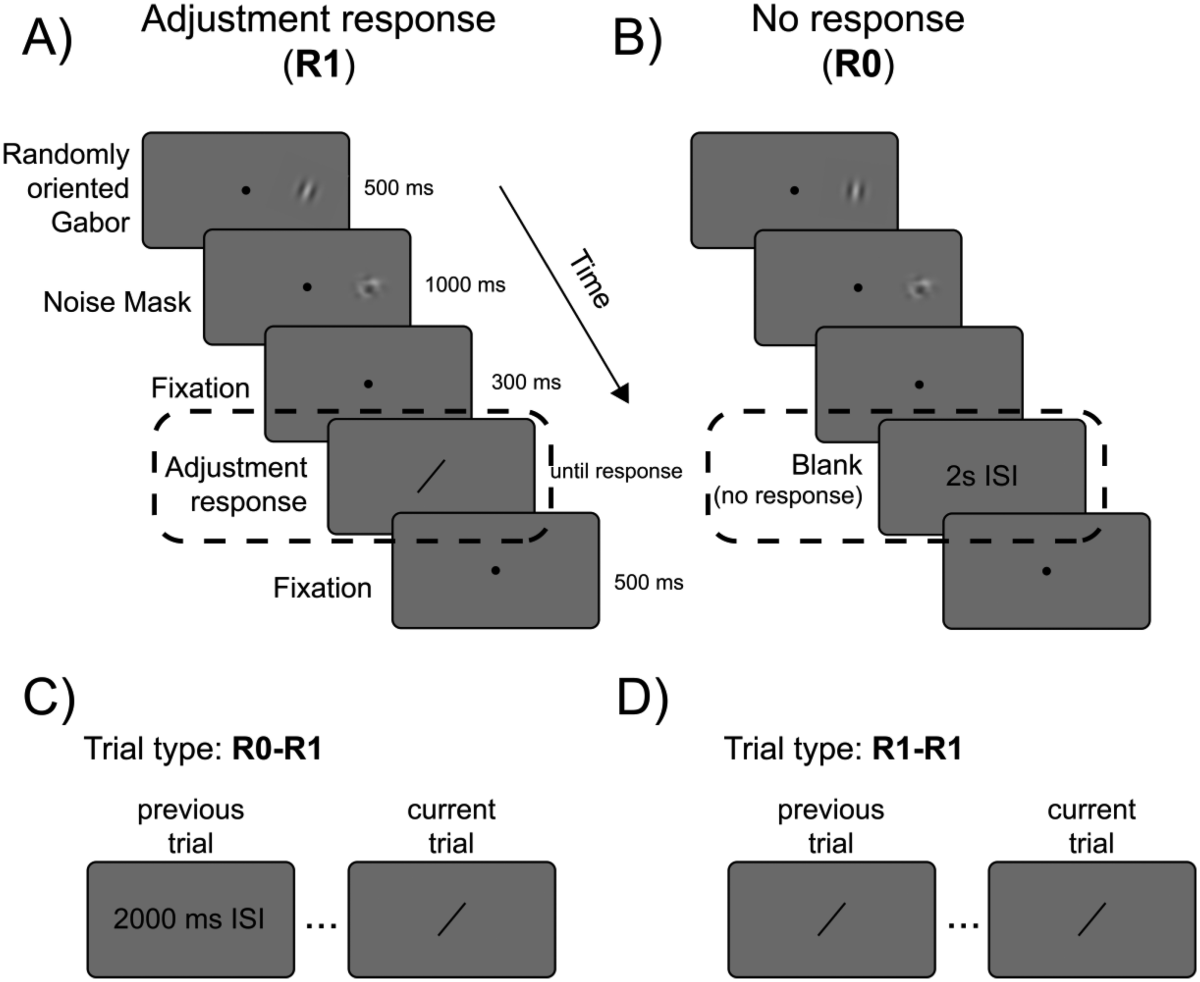
The sequence of stimuli in the two types of trials. In both trials, participants were instructed to fixate the central dot. (A) A Gabor stimulus was presented in the right visual field for 500 ms, followed by a noisy mask for 1000 ms. (B In R1 trials, a response bar appeared at the center of the screen after 300 ms and participants had to rotate the bar to match the perceived orientation of the stimulus (left panel). (C) In R0 trials, the response bar was not presented and replaced by a blank interval of 2 s. (D) For the analysis of serial dependence, the trial type R0–R1 indicates the condition in which the previous trial contained no response. R1–R1 indicates the condition in which the previous trial contained a response.

Here, we tested serial dependence in a classic orientation adjustment task^25,26,33–41^ in 26 SCZ patients and 27 CNT. The task required participants to adjust a response bar to match the perceived orientation of a stimulus (figure 1). As mentioned, we choose the orientation adjustment task as it is a well-established paradigm in which healthy CNT typically show robust attractive biases—responses tend to be biased toward the orientation of the preceding stimuli, indicating the integration of prior stimuli with the sensory information of current ones.^27,28^ Our study sought to provide complementary evidence of reduced serial dependence in SCZ patients using distinct paradigms and stimuli.

We compared key aspects of serial dependence, including the typical effect of the preceding stimulus, the effect of trials involving or not a response, the temporal “tuning” of serial dependence, that is, the persistence of the effect for several stimuli in the past and its feature “tuning,” and the finding that serial dependence occurs for more similar sequential objects but not for dissimilar ones.^26,28,42^ Consistent with previous findings, we observed robust serial dependence in the healthy CNT group, with effects extending up to three trials in the past. In the SCZ group, we found intact serial dependence with a strength comparable to that of the CNT group. These findings challenge the general notion of reduced serial dependence and the assumption of a generalized reduction in the influence of prior stimuli in SCZ, calling for a more comprehensive assessment of different paradigms and stimuli.

## Methods

### Participants

Twenty-seven healthy CNT and 26 SCZ patients participated in the study. All participants had normal or corrected-to-normal vision, with visual acuity >0.8 (corresponding to 20/25) at least in one eye, as determined by the Freiburg Visual Acuity test.^43^ Four patients and one healthy CNT were excluded because they were unable to perform the task (see Data analysis).

Healthy CNT were recruited from the general population, aiming to match the demographic characteristics (gender, age, and education) of the patients as closely as possible. All CNT were free from psychiatric axis I disorders and had no family history of psychosis. The general exclusion criteria were drug or alcohol abuse, and neurological or other somatic illnesses influencing participants’ mental states. Participants were no older than 55 years. Ethics approval was obtained in Tbilisi from the Georgian National Council on Bioethics.

SCZ patients were recruited from the Tbilisi Mental Health Hospital or the psycho-social rehabilitation center. Patients were diagnosed according to DSM-IV, by means of an interview based on the SCID, information from the staff, and the study of the records. The psychopathology of SCZ patients was assessed by an experienced psychiatrist using Scales for the Assessment of Negative Symptoms and Scales for the Assessment of Positive Symptoms (SANS, SAPS; Andreasen, 1984, 1989). Patients were invited to participate in the study when they had sufficiently recovered from the acute psychotic episode. Out of the 26 patients, only two were not receiving neuroleptic medications. Three were inpatients, and 23 were outpatients. Group characteristics and chlorpromazine (CPZ) equivalents are indicated in table 1. All participants signed informed consent and were informed that they could quit the experiments at any time.

**Table 1.** Demographic Data (mean ± standard deviation) of CNT and SCZ patients

Participants	Healthy Controls (CNT)	Schizophrenia (SCZ) Patients
*N*	27	26
Age (years) ± SD	37.3 ± 8.30	38.11 ± 9.28
Gender (f/m)	7/20	8/18
Education (years) ± SD	15.70 ± 2.26	13.88 ± 2.68
Illness duration (years) ± SD		13.57 ± 9.88
SANS ± SD		9.11 ± 4.58
SAPS ± SD		8.19 ± 2.87
CPZ ± SD		576.58 ± 408.38

It is important to note that the same participants underwent a more extensive battery of tests, showing strongly deteriorated performance in the Continuous Performance Test, the Wisconsin Card Sorting Test, and visual backward masking.^44,45^ In addition, patients show strongly diminished EEG amplitudes and varied microstates,^46,47^ which are specifically associated with psychosis.^48^ Therefore, the two groups were representative of distinct populations of interest, and patients demonstrated clear and typical visual impairments in vision.

### Apparatus

The stimuli were presented on an LCD screen (ASUS VG248QE, Taipei, Taiwan; screen resolution 1920 × 1080 pixels). The refresh rate was 100 Hz. All experiments were programmed in MATLAB (The MathWorks, Natick, MA) using the Psychophysics Toolbox.^49^ Stimuli were presented on a gray background and were viewed from a distance of 57 cm. The experimental room was dimly illuminated. Participants used a keyboard for all responses (left-right arrow keys to adjust the bar, and a space bar to confirm bar orientation and initiate the next trial).

### Stimuli and Procedure

During the entire experiment, a 0.2° diameter white dot served as a fixation point. Subjects were instructed to always maintain fixation while performing the task. We presented a randomly oriented Gabor at 10° of eccentricity in the right visual field. The Gabor (windowed sine-wave gratings) had a peak Michelson contrast of 80%, a spatial frequency of 0.5 cycles per degree, and a 0.9° SD Gaussian contrast envelope with Brownian noise (1/f^2^ spatial noise). Gabors were presented for 500 ms, after which a noise mask was presented for 1000 ms at the same location. Noise patches consisted of white noise smoothed with a 0.85° SD Gaussian kernel and windowed in a 0.9° SD Gaussian contrast envelope and were presented to minimize negative aftereffects.

After a 300-ms delay, two kinds of trials were presented. In trial R1 (figure 1), a response bar (width: 0.5°, length: 4°, color: dark gray) appeared at the fixation point location. Participants were asked to adjust its orientation to match the perceived orientation of the Gabor using the left/right arrow keys. The starting orientation of the bar was randomized for each trial. The observers were asked to adjust the bar as fast as possible and to press the spacebar to confirm the chosen bar orientation. In trial R0 (figure 1), no response bar appeared, and subjects were asked to keep fixating the dot with no response for 2 additional seconds. After a 500-ms delay, the next trial started. The two types of trials were randomly intermixed. Within a single block (50 trials each), trials R1 and R0 were 35 and 15, respectively. Trials without a response (R0) were included to measure serial dependence in the absence of any confounding due to the response tool and to ensure an equal time interval between Gabors, independent of the adjustment time of the participant and group. The main conditions of interest are, therefore, indicated as R0–R1, when the previous trial contained no response, and R1–R1, when the previous trial contained a response.

The experiment was composed of four sessions, divided into four blocks each (16 blocks, 800 trials in total). To familiarize themselves with the stimuli and procedure, subjects performed a practice session consisting of four blocks (20 trials each, 80 trials in total).

### Data Analysis

#### Preprocessing

At the level of individual trials, we removed outliers in a two-step procedure. First, adjustment errors were computed because the acute angle between the reported and true orientation, in degrees, according to:


$$\begin{array}{l} error={\left((r-\theta )+90\right)}_{mod180{}^{\circ }}-90 \end{array}$$

where *r* is the reported orientation, θ is the actual stimulus orientation, and $mod180{}^{\circ }$ indicates the application of the modulo operation with a divisor of 180° to ensure that the resulting error values fall within the range of −90 to 90°.

Absolute errors larger than 45° were considered lapses and removed. The remaining errors were demeaned and then systematic biases in reporting orientations were residualized^35^ by eliminating the fit of a sum of six sinusoids^35^ using the MATLAB function *fit.m* with the model specification “sin6.”

In a second step, errors were then cleaned from outliers identified as values more than 1.5 interquartile ranges above the upper quartile or below the lower quartile (function *isoutlier.m* with method = “quartiles” in MATLAB). Adjustment trials slower than 4 s were also excluded from further analysis. Less than 15% of trials were excluded in total (11% for the CNT group and 13% for SCZ group).

At the level of participants, we excluded all data from participants with a standard deviation of adjustment errors larger than 35° and with more than 25% of outlier trials (one excluded in the CNT group and four in the SCZ group). The chosen cutoff value of 35° corresponds approximately to the value of the standard deviation reached by a simulated observer providing random responses on 50% of the trials.

**Fig. 2.**
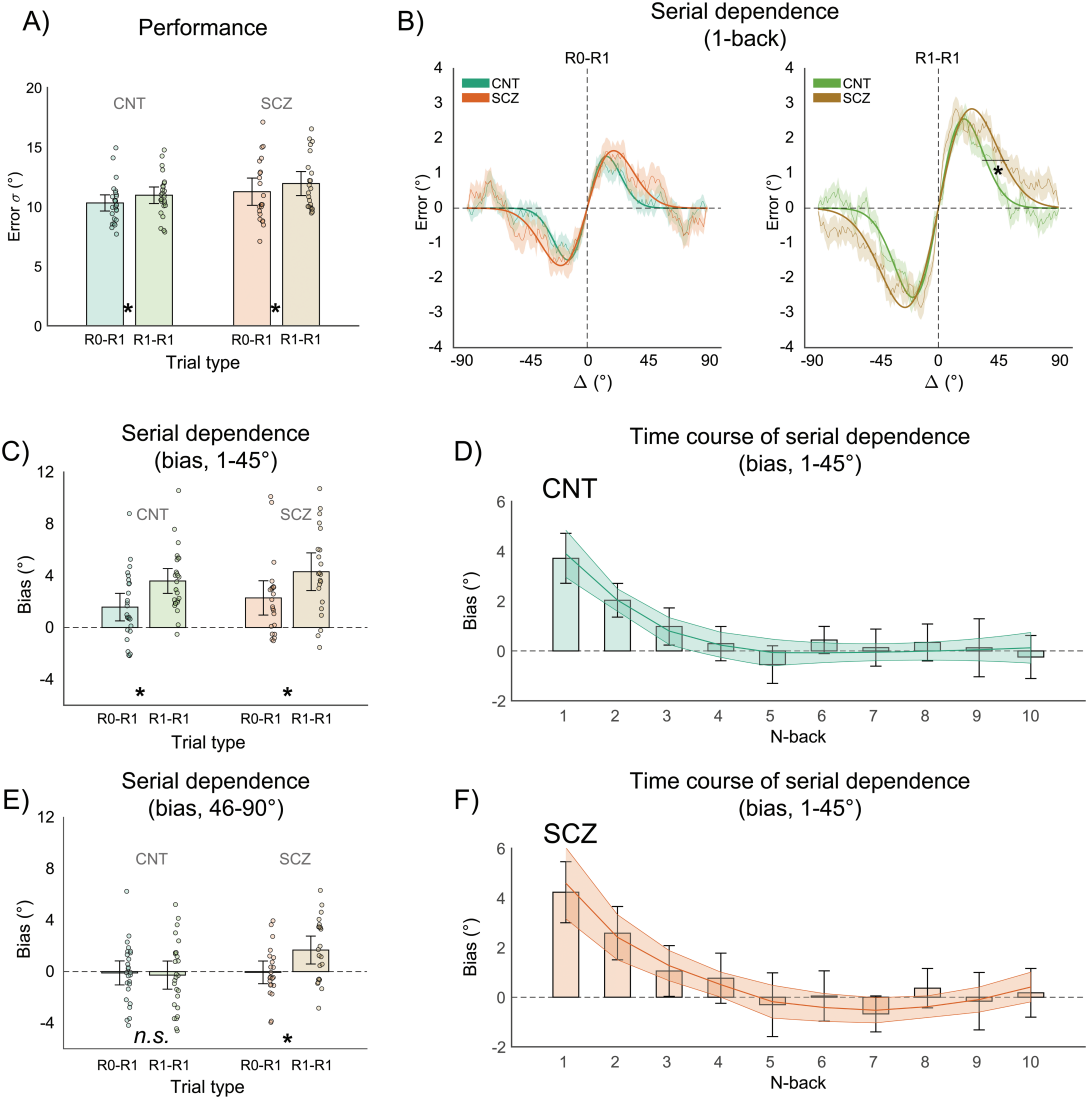
(A) Performance in the orientation adjustment task, as measured by the error SD (Error σ) for both the control (CNT) (bars in the green color range) and schizophrenia (SCZ) groups (bars in the red color range), and for trials after no-response (R0–R1) and after response (R1–R1). (B) Serial dependence curves and δoG fit for the R0–R1 condition (left panel, green lines and shaded areas are for the CNT group, orange lines and shaded areas are for the SCZ group) and the R1–R1 condition (right panel, green lines and shaded areas are for the CNT group, brown lines and shaded areas are for the SCZ group). Error bars are 95%CI. A moving average of the aggregate errors of all participants in each group is plotted on the y axis, as a function of the difference between the previous and current orientation (Δ, previous minus current). Lines are best-fitting δoG curves. Shaded areas represent 1 SEM of the moving average. (C–E) Control analysis of serial dependence, computing the bias for the Δ range 1–45° (C) and 46–90° (E). The color coding is the same as in (A). (D–F) Time course of serial dependence shown by the change of the bias (Δ range = 1–45°) as a function of 10 stimuli in the past. The fitting line and shaded areas reflect the fit of a segmented regression with 95%CI.

As a measure of performance in the orientation adjustment task, we computed the error dispersion (the standard deviation of adjustment errors, *std.m* in MATLAB, or Error σ, figure 2A). Overall, the CNT group performed the adjustment task with average adjustment times of 1.92 s, whereas the SCZ group performed the adjustment task with average adjustment times of 2.02 s.

#### Analysis of Serial Dependence

Serial dependence in adjustment responses was analyzed using a model-based approach^26^ and a control analysis based on an arbitrary binning.^38,50^ The model-based analysis consisted of fitting a first derivative of a Gaussian function^26^ to the adjustment errors as a function of the variable Δ, obtained as previous minus current orientation:


$$\begin{array}{l} \;\Delta \;={\left(\left(\theta_{n-1}-\theta_{n}\right)+90\right)}_{mod180{}^{\circ }}-90 \end{array}$$

where $\theta_{n-1}$ and $\theta_{n}$ are the orientations of the stimulus shown on the preceding (n−1) and current trial (*n*), respectively. The δoG has the following form:


$$\begin{array}{l} error=\Delta \alpha wce^{-{\left(w\Delta \right)}^{2}} \end{array}$$

where $c=\sqrt{2}/e^{-0.5}$ is a constant and *w* is the inverse of the curve width. The half-amplitude parameter α quantifies the deviation of the errors, in degrees, from the actual orientation as a function of the Δ variable: positive values of α indicate a systematic deviation of errors toward the orientation of the preceding stimulus, and negative values indicate a deviation away—ie, repulsion. The parameters of the δoG function were estimated on the aggregate data of all participants, by solving a constrained nonlinear minimization problem with the sum of squared residuals as the cost function (using *fmincon.m* in MATLAB). The initial parameters were set to α=2, w=0.05, and were refined through a search grid. The search constraints (upper and lower bounds) were α=[−20, +20],  w=[0.01, 0.1].

Model fitting was performed separately for each group (CNT and SCZ) and trial sequence (R1–R1 or R0–R1). This statistical significance of the half-amplitude and width parameters was assessed via bootstrap resampling and surrogate null statistics, by randomly shuffling the sign of adjustment errors and comparing the observed parameters with the distribution of surrogate ones (*N* = 10 000). Serial dependence between groups and conditions was compared by randomly shuffling the group/condition labels 10 000 times and comparing the distribution of the resulting differences against the observed ones. In figure 2B, the average curves are depicted using folded errors, calculated by multiplying trial-wise errors by the sign of the trial-wise Δ. This approach is in line with recommendations from previous studies.^51^ Given the symmetric nature of serial dependence patterns, folded errors for negative Δ are therefore represented as a mirrored and sign-flipped version of those in the positive range. It is important to note that this procedure is solely for graphical purposes, and all analyses, including model fit, were performed on the original error variable.

In the arbitrary binning approach, we subtracted the average error for positive Δ values from the average error for the corresponding negative Δ values.^38,42,50^ In order to characterize effects spanning the entire range of Δ, we separately considered two Δ ranges, corresponding to small (1–45°) and large orientation differences between the current and previous stimulus (46–90°). The 45° interval was chosen based on prior work,^38,50^ and on the rationale that serial dependence is typically positive at small orientation differences (eg, <45°^28^) and null or even repulsive at larger differences(eg, >45°^52^). The resulting index, quantifying the amount of systematic deviation of the errors from zero (either in the positive or negative direction), was used for subsequent analysis.

In the Bayes Factor (BF) analysis, we used the attractive bias in the small Δ bin, collapsing the trial type, and estimated the average bias in R0 and R1 trials, separately for each group. We then calculated the JZS BF (scale parameter = 0.707) for a two-sample *t* test (BF_01_, quantifying evidence in support of the null hypothesis, ie, of no difference in bias between groups). Additionally, we also estimated the BF_10_ (evidence in favor of the alternative hypothesis) for the presence of serial dependence biases in both groups. Complementary to this, we conducted a bootstrap analysis using a resampling technique. We generated 1 000 000 bootstrap samples for each group by randomly selecting, with replacement, individual bias values from the original data, in the small Δ bin. For each bootstrap sample, we computed the difference in means between the two groups and calculated the 2.5th and 97.5th percentiles of the bootstrap differences to establish a 95%CI.

An estimate of the bias derived in a similar way was also used in the analysis of n-back serial dependence, where Δ was computed considering stimuli shown in up to 10 trials in the past. This analysis served to evaluate and compare the time course of serial dependence between groups. Because serial dependence was larger, in both groups, for small Δs, we restricted this analysis to the bias in the small orientation difference range (1–45°). The bias computed with respect to the orientation of stimuli in the preceding 10 trials was modeled with a segmented regression of the form:


$$\begin{array}{l} \begin{array}{lll} & \begin{array}{lll} y=\beta_{0}+\beta_{1}x & \;\;for\;\;\;x<b\;y=\beta_{2}+\beta_{3}x & \;\;for\;\;\;x>b\; \end{array}\; & \;\;\;\;\;\;\;\;\;\;\;\;\;\;\; \end{array} \end{array}$$

yielding five parameters: (1) the starting point of the bias due to the stimulus shown in one trial in the past ($\beta_{0}$), (2) the slope of the bias as a function of more stimuli in the past ($\beta_{1}$), (3) the “breakpoint” at which the initial effect reaches zero (*b*), and (4–5) the intercept ($\beta_{2}$) and slope ($\beta_{3}$) of a second segment after the breakpoint. These parameters were individually estimated for each participant using the *fminbnd.m* optimization routine in MATLAB, which searched for the optimal breakpoint to minimize the residual norm between the observed and predicted values. Subsequently, a linear regression was applied to estimate intercepts and slopes at the two segments. The estimated parameters were then compared between groups using a *t* test.

## Results

We first investigated whether SCZ patients performed worse in the orientation adjustment task than healthy CNT. To this end, we analyzed the SD of the adjustment errors (Error σ) in a split-plot ANOVA with group (CNT vs SCZ) as the between-subject factor and trial type (R0–R1 vs R1–R1) as the within-subject factor. The results revealed a significant main effect of the previous trial type (*F*(1,46) = 23.48, *P* < .001) but neither a main effect of group (*F*(1,46) = 2.72, *P* = .105) nor an interaction (*F*(1,46) = 0.01, *P* *=* .89), indicating comparable performance between patients and CNT (see figure 2A). There was no difference in the average adjustment times between groups as well (CNT = 1.92 + −0.25 s, SCZ = 2.02 + −0.28 s, difference =  *t*(46) = −1.32, *P* = .194, two-sample *t* -test).

Fitting of the δoG function revealed serial dependence in all conditions, as indicated by peaks of the bias greater than 1° (eg, half-amplitude values, α, see figure 2B). In the condition without a response on the previous trial (R0–R1), there was no difference across groups, both in the peak (1.31° for CNT, 1.53° for the SCZ, difference = −0.22°, *P*_*perm*_  = .307) and width (0.04 for CNT, 0.03 for SCZ, difference = 0.012, *P*_*perm*_  = .141) of the fit. In the condition with a response on the preceding trial (R1–R1 condition), the peak of serial dependence was comparable between groups (2.40° for CNT, 2.73° for SCZ, difference = −0.33°, *P*_*perm*_ = .210), but the width was different, indicating a broader effect of prior stimuli in SCZ (0.03 for CNT, 0.025 for SCZ, difference = 0.008, *P*_*perm*_ = .025; figure 2B, right panel).

Additionally, the presence of a response (and related adjustment bar) on the preceding trial had a clear effect in both groups, leading to nearly doubled serial dependence (CNT: *α*[R0–R1] = 1.31°, *α*[R1] = 2.40°, difference = −1.08°, *P*_*perm*_  = .008; SCZ: *α*[R0] = 1.53°, *α*[R1–R1] = 2.73°, difference = −1.19°, *P*_*perm*_  = .008; no significant difference in width, all *P*_*perm*_* *> .05).

These results, obtained via a fitting procedure on the aggregate data, were fully supported by a control analysis based on arbitrary binning (see Methods) that considered interindividual variability. In this analysis, we conducted a split-plot ANOVA on a measure that quantifies the bias toward previous orientations. Two separate ANOVAs were performed based on the magnitude of the absolute difference in orientation between the current and previous stimuli, distinguishing between small and large differences (see Methods). Each ANOVA included the variable Group (CNT vs SCZ) as the between-subject factor and the previous trial type (R0–R1 vs R1–R1) as the within-subject factor. For small orientation differences (Δ = 1–45°), the ANOVA revealed a significant main effect of the previous trial type (*F*(1,46) = 16.66, *P* < .001), but no main effect of Group (*F*(1,46) = 1.21, *P* = .276) and no interaction between Group and previous trial type (*F*(1,46) < 0.001, *P* = .996). For larger orientation differences (Δ = 46–90°), the ANOVA revealed no significant main effect of Group (*F*(1,46) = 3.57, *P* = .064) or main effect of the previous trial type (*F*(1,46) = 3.00, *P* = .089), but a significant interaction between the two (*F*(1,46) = 4.47, *P* = .039). Post hoc testing indicated that the interaction was driven by an increase in bias in the R1–R1 condition compared with the R0–R1 condition, specifically in the SCZ group (*t*(21) = 2.34, *P* = .029, paired *t* test, Cohen’s *d’* = 0.499), while no such difference was observed in the CNT group (*t*(25) = 0.31, *P* = .754). Thus, in line with the model fitting results, both groups exhibited comparable serial dependence and comparable effects of the previous response, with a slight increase in serial dependence following trials with a response in the SCZ group.

Following the results presented above, we estimated the BF and 95% bootstrap CI of the main effects of interest, namely, the presence of attractive serial dependence at small Δ in both groups and the difference between groups (see Methods). This resulted in extreme evidence of attractive serial dependence biases in both groups (BF_10_ > 100), with 95%CI of the effects largely separated from zero (CNT = [1.8810, 3.3110], SCZ = [2.2992, 4.3953]), and anecdotal evidence in support of the null hypothesis of no difference between groups (BF_01_ = 2.119; 95%CI = [−2.0150, 0.5299], mean bootstrap difference = −0.7126°, in the direction of larger biases in SCZ). The width of the 95%CI of this difference (2.5415) was comparable to the one obtained via permutations, by randomly shuffling the group label (2.5223).

Finally, we analyzed the time course of serial dependence. This analysis was performed to evaluate whether patients and CNT would not only show a comparable effect of the immediately preceding stimulus but also comparable temporal dynamics of serial dependence. In this analysis, we examined the bias averaged within the small Δ range (1–45°), where the effects of serial dependence were the strongest, and assessed the bias for up to 10 trials in the past. For each participant in each group, we performed a segmented regression to model the bias as a function of the n-back trials (see Methods). Both the CNT and SCZ groups showed a significant intercept (CNT = 5.81°, *t*(25) = 6.33, *P* < .001; SCZ = 7.07°, *t*(21) = 6.03, *P* < .001) and slope (CNT = −1.91°, *t*(25) = −3.93, *P* < .001; SCZ = −2.47°, *t*(21) = −4.29, *P* < .001) of a segment starting at 1-back and reaching zero at approximately four trials (CNT = 4.37, *t*(25) = 15.70, *P* < .001; SCZ = 4.76, *t*(21) = 10.02, *P* < .001), indicating effects up to three trials in the past, consistent with previous findings.^26^ The intercept, slope, and breakpoint of this segment did not significantly differ between the groups (intercept: *t*(46) = −0.85, *P* = .396; slope: *t*(46) = 0.74, *P* = .458; breakpoint: *t*(46) = −0.71, *P* = .475). The intercept and slope of a second segment starting from the 4th and extending to the 10th trial back were not significantly different from zero in the CNT group (intercept = −0.54°, *t*(25) = −0.74, *P* = .464; slope = 0.07°, *t*(25) = 0.71, *P* = .481), and nearly significant in the SCZ group after a Bonferroni correction of 0.05 with five comparisons (eg, *P* < .01) (intercept = −4.46°, *t*(21) = −2.82, *P* = .010; slope = 0.50°, *t*(21) = 2.81, *P* = .010), but did not differ between groups (all *P* > .05, Bonferroni corrected). Hence, the temporal decay of serial dependence was also comparable between the two groups.

## Discussion

We investigated serial dependence in 27 healthy CNT and 26 individuals with SCZ using an orientation adjustment task. Consistently with previous research, we found strong serial dependence in healthy CNT, with effects extending up to three trials in the past (~10–15 s^26,28^). Our results show intact serial dependence in SCZ as well. Such findings challenge the notion that reduced serial dependence is a universal characteristic of the disease, suggesting instead that deficits in integrating prior stimulus information may be specific to certain paradigms and stimuli.

Our results seem to be at odds with recent evidence reporting reduced serial dependence in SCZ.^32^ In the study by Stein et al., participants performed a visuospatial memory task where they reproduced the location of a stimulus after varying delays. They found that only the CNT group exhibited serial dependence, and only in one condition (at long stimulus-response delays of 3 s). The SCZ group, along with a third group of patients with autoimmune anti-NMDAR encephalitis, and the CNT group at shorter delays, displayed only strongly reduced or no attractive serial dependence. More importantly, the SCZ group showed clear repulsive effects, indicating that responses were biased away from, rather than attracted toward prior stimuli, and this effect increased with longer stimulus-response delays. Based on these findings, the authors concluded that a specific disruption in long-lasting memory traces in SCZ prevents the integration of prior stimuli with current perception.^32^ Similar findings were reported in a recent study by Bansal et al.^31^ and Gold et al.^30^ The critical factor in these studies seems to be the delay between the stimulus and the response, with attractive (in healthy CNT) or repulsive serial dependence (in patients) becoming evident only at longer delays.

In our study, we did not manipulate the stimulus-response delay, which may be considered a limitation. However, it is crucial to highlight that we observed intact serial dependence in SCZ, without clear repulsive effects, using a classic paradigm where attractive biases are typically evident even at short stimulus-response delays.^28^ Moreover, prior studies did not consider the effect of response trials, which is a crucial variable in serial dependence. Differences between groups could arise merely from differences in response times influencing the interval between stimuli. Here we adopted a rigorous approach where the intertrial interval was equated across groups (in the R0–R1 condition) to ensure that differences in response times were not a factor at play.

One possible explanation is that the repulsion previously reported reflects abnormally heightened adaptation and negative aftereffects in SCZ, as demonstrated in studies on the tilt-aftereffect.^53–55^ In earlier studies, stimuli were unmasked and highly visible (eg, high contrast), conditions known to enhance sensory adaptation and induce repulsive effects, ultimately leading to diminished serial dependence.^56^ Consequently, due to heightened adaptation, patients might exhibit more pronounced repulsive effects, overshadowing the attractive bias typically associated with serial dependence, considering that these two effects coexist and compete.^35,57,58^

The exact mechanisms regulating the interplay between sensory adaptation and attractive serial dependence in SCZ go beyond the scope of our study, as our aim was to assess serial dependence in a paradigm where adaptation effects are minimized. To achieve this aim, we used low-contrast stimuli and masks, two manipulations that can yield specific effects: low-contrast stimuli mitigate adaptation at the sensory level, while the high-contrast mask, lacking specific feature information, may uniformly redistribute any residual adaptation across neurons sensitive to different features. The resulting absence of repulsive effects suggests that the choice of stimuli and experimental design are crucial factors in determining the strength of repulsive and attractive biases. We propose that future research should investigate the interplay between sensory adaptation and attractive serial dependence under varying stimulus parameters, manipulating contrast, masking, and stimulus-response delay to provide a more explicit test of the suggested mechanisms and their disfunction in SCZ. This includes, for instance, evaluating the predictions of mechanistic models, such as those based on NMDAR hypofunction, which have been proposed to explain both reduced serial dependence and heightened adaptation in SCZ.^32,54^

Overall, the work by Stein et al., Bansal et al., Gold et al.,^31,32^ and our study contribute to demonstrating that, in the context of serial dependence, deficits in integrating priors in SCZ may not be a universal characteristic of the disease. In particular, repulsion and delay-related effects might manifest in tasks with clear and strong sensory signals, involving delayed working memory maintenance, while attractive serial dependence may prevail in tasks minimizing adaptation effects and tapping into perceptual decision-making under uncertainty.

It must be noted that mixed results have also been found using different tasks, like those involving forced choice responses and choice history biases. For example, Eckert et al.^59^ and Lieder et al.^60^ investigated history biases in psychosis-prone individuals and individuals with autism, respectively, and reported reduced effects of the immediate past on perceptual decisions compared to healthy CNT. In contrast, other studies have found no difference with CNT,^61^ or even an increased influence of prior choices in autism.^62^ These studies have also used different stimuli and designs, suggesting again that deficits may be specific to certain tasks and stimulus parameters.

Regarding statistical power, it is important to note that previous studies and a meta-analysis^27,28^ indicate that effect sizes for biases in typical serial dependence tasks, similar to the one employed in this study, fall within the medium-to-large range (Cohen’s *d’* > 0.6). Such effect sizes require smaller sample sizes than the one utilized in our study to achieve 80% power (eg, a sample size of *N* = 19 would be sufficient to detect a significant bias against 0, assuming a minimum effect size of *d’* = 0.6, α crit = 0.05, and power = 80%, as per G*Power results). Additionally, concerning differences between the effects observed in the SCZ and CNT groups, prior research has reported substantial effect sizes (eg, *d’* = 2.21 in Stein et al.^32^). If similar differences exist in our paradigm, the necessary sample size to detect them with 80% power would be relatively small (eg, five subjects per group would be adequate to identify a significant difference between independent groups at 80% power, according to G*Power results). Therefore, our results are not simply due to measurement errors and sample size.

In our experiment, we did not record eye movements. This raises the possibility that differences in eye position could contribute to the lack of differences between the two groups. For instance, one might argue that patients fixated more frequently on the stimulus, thus leading to an orientation discrimination performance comparable to the CNT, as orientation resolution is higher at the fovea. However, this potential confounding does not impact serial dependence for several reasons. Firstly, if patients had consistently foveated on the stimulus instead of maintaining fixation, this would not necessarily result in differences in serial dependence. Previous research indicates that the strength of serial dependence is similar whether the stimulus is presented at the fovea or in the periphery.^35,39^ Secondly, even if patients had more frequently moved their eyes toward the stimulus or toward random locations during the experiment, the observed serial dependence would likely still be comparable to that of the healthy CNT group, as studies have shown that serial dependence persists even when the location of the current and previous stimulus changes by 15–20°.^28^ Therefore, differences in eye movements are unlikely to account for the comparable levels of serial dependence.

Both the healthy CNT and SCZ groups exhibited significant serial dependence without a response, a finding that replicates previous studies in typical populations,^26,33,41,56,63–68,see27^^for a review^ and indicates that motor responses are not necessary for serial dependence. Nevertheless, serial dependence was significantly stronger following trials with a response, a finding also reported in previous studies.^35,40–42,56,69^ Several factors in our paradigm could contribute to the stronger effect with responses, including (1) an additional orientation cue (eg, response bar) in the R1–R1 condition, potentially amplifying the serial dependence effect; (2) additional attention devoted to the same or similar orientation information, which increases serial dependence^28^; and (3) top-down modulation of serial dependence due to memory and decision-making processes related to the response stage.^35^ Considering the latter possibility and the hypothesis that individuals with SCZ exhibit diminished influence of priors at higher, top-down levels of the processing hierarchy,^24^ one might expect a reduced effect of response manipulation in SCZ. However, our results demonstrate that this aspect is intact in patients as well.

Furthermore, not only the strength of the effect of the previous stimulus and the impact of previous responses but also the temporal dynamics of serial dependence were comparable between healthy CNT and patients. Specifically, both groups exhibited a decrease in the impact of prior stimuli after three to four trials, which aligns with findings from previous studies and the typical temporal tuning of these effects.^26,28^ Interestingly, our analyses on feature tuning of serial dependence unexpectedly revealed an even broader bias in patients compared to CNT. Particularly, after response trials (although a similar trend can be observed after no-response trials as well, as depicted in figure 2B, first panel), the bias toward prior stimuli extended to a slightly but significantly wider range of orientations in the SCZ group (see the x-axis peak in figure 2B).

Taken together, this body of results reinforces the idea of serial dependence as a pervasive,^27,28^ stable,^39^ and general attractive bias evident across different populations.^60,61,70^ Our findings emphasize the importance of reporting comparable performance between patients and CNT in SCZ research. Typically, significant differences suggest abnormal performance in patients. However, when patients perform on par with CNT and these results go unpublished, it can erroneously create the impression that patients consistently perform differently in all paradigms, which is not the case.

Another related aspect is generalization. Paradigms are often considered representative of specific types of processing.^71^ However, when we compare the results of our paradigm with those of Stein et al.^32^ and Bansal et al.,^31^ it becomes evident that this assumption does not always hold true. A deficit observed in one paradigm may not extend beyond the particular experimental setup and stimuli used, providing limited insights into general cognitive functions.^23^ Therefore, the serial dependence observed for orientation in our study may not necessarily be indicative of other forms of serial dependence or the use of priors in SCZ more broadly. Nonetheless, our findings serve as a starting point and establish a “baseline” condition in which patients perform similarly to CNT.

In conclusion, our study reveals intact serial dependence in visual orientation processing among individuals with SCZ. These findings open the door for future investigations into potential differences in higher-level perceptual and cognitive stages, encompassing a broader range of stimuli. For instance, future research could test serial dependence with more complex stimuli, such as faces and emotions,^67,72^ providing a comprehensive assessment of whether and at which processing stage patients may exhibit different use of priors compared to CNT.

## Data Availability

The raw data and analysis code will be made available in public repositories upon acceptance.
